# Nimodipine after aneurysmal subarachnoid hemorrhage: shortened treatment in an unselected patient cohort

**DOI:** 10.1016/j.bas.2025.104376

**Published:** 2025-07-28

**Authors:** Bryndís Baldvinsdóttir, Peo Wästberg, Björn M. Hansen, Erik Uvelius, Erik Kronvall

**Affiliations:** a Department of Clinical Sciences, Neurosurgery, Lund University, Lund, Sweden; b Department of Clinical Sciences, Radiology, Lund University, Lund, Sweden

**Keywords:** Subarachnoid hemorrhage, Nimodipine, Delayed cerebral ischemia, Intracranial aneurysm, Vasospasm

## Abstract

**Background:**

Nimodipine improves outcome after aneurysmal subarachnoid hemorrhage (aSAH) through mitigation of delayed cerebral ischemia (DCI). Most studies are based on a treatment duration of 21 days. At our institution, clinical practice is to administer nimodipine for 14 days, regardless of bleeding severity. Treatment is prolonged if signs or symptoms of DCI occurs. The present study aims to review this practice.

**Methods:**

A prospective cohort of aSAH patients was reviewed and relevant information regarding nimodipine treatment were retrospectively added to this database. Functional outcome was measured using Glasgow outcome scale extended (GOSE) one year after ictus and dichotomized into unfavorable and favorable outcome. Radiological outcome was defined by the occurrence of new cerebral infarctions on brain imaging later than 30 days post-ictus.

**Results:**

The study population comprised 164 patients, out of which 97 (59 %) received nimodipine for 14 days or less. Unfavorable outcome was noted in 27 % of patients and brain imaging found cerebral infarctions in 17 % of patients. Both outcome measures were similar to previously published studies. No readmissions or signs of DCI were seen after discontinuation of nimodipine.

**Conclusions:**

A shortened nimodipine treatment period in patients without DCI after aSAH could be feasible. This may reduce sleep deprivation of patients and more effective utilization of neurointensive care resources. A large, randomized study is required to answer the question whether a shorter treatment with nimodipine is adequate to give full benefit of the medication in patients without signs and symptoms of DCI.

## Highlights

• Nimodipine reduces the risk of delayed cerebral ischemia following aneurysmal subarachnoid hemorrhage.
• Most guidelines recommend nimodipine treatment for 21 days.
• In Lund, Sweden, nimodipine is administered for 14 days but the treatment is prolonged if clinically indicated.
• Majority of patients received nimodipine treatment for 14 days.
• Functional outcome was comparable to previously published studies.

## 1 Introduction

Aneurysmal subarachnoid hemorrhage (aSAH) is often devastating with ensuing high morbidity and mortality (Hoh et al., 2023; Wahood et al., 2022; Macdonald, 2021). Patients with aSAH are at risk of developing delayed cerebral ischemia (DCI) within the first weeks. DCI is defined as clinical neurological deterioration or cerebral infarction on imaging, without any other reasonable explanation (Hijdra et al., 1986; Vergouwen et al., 2010). Patients who develop DCI are at higher risk of death and poor functional recovery (Hoh et al., 2023; Vergouwen et al., 2010; Eagles et al., 2019; Geraghty and Testai, 2017; Khanafer et al., 2022). Treatment with the calcium channel inhibitor nimodipine has been shown to improve outcome after aSAH and reduce the risk of developing DCI (Dorhout Mees et al., 2007; Allen et al., 1983; Pickard et al., 1989).

The trials introducing nimodipine in the care of aSAH patients were conducted with the hypothesis that vasospasm was the sole underlying cause of DCI (Locksley, 1966; Dodd et al., 2021). Given that vasospasm is most prevalent during the initial three weeks after aSAH, this treatment length was used in the trials evaluating nimodipine (Allen et al., 1983). Therefore, most international guidelines recommend a 21-day nimodipine treatment for all patients, regardless of aSAH severity (Diringer et al., 2011; Vivancos et al., 2014; Treggiari et al., 2023; Protocol for Administration of Nimodipine, 2009). More recent studies have shown that patients with mild or no vasospasm may develop DCI, and that patients with severe vasospasm may not. This suggests a more complex pathophysiology (Geraghty and Testai, 2017; Macdonald et al., 2007). Additionally, there is no evidence that systemic nimodipine reduces the frequency or severity of vasospasm (Locksley, 1966; Dodd et al., 2021). Recent research has demonstrated that nimodipine has both neuroprotective and neuroregenerative properties (Bork et al., 2015).

As nimodipine likely acts through other mechanisms than directly counteracting vasospasm, the basis for the 21-day treatment period can be questioned. Two studies have investigated whether 21-day treatment is necessary to gain the full benefits of nimodipine (Cho et al., 2016; Sokolowski et al., 2021). No significant differences in mortality or functional outcome was seen based on treatment length. However, these findings were mainly based on good clinical grade aSAH patients.

In our department, standard practice is to administer nimodipine for 14 days, with the option to prolong treatment if clinically indicated, even in patients who are initially in a poor clinical state. The current study presents clinical characteristics and one year outcome in an unselected cohort of aSAH patients, aiming at reviewing the safety and applicability of this treatment regime.

## 2 Methods and materials

The study population is based on an unselected prospective cohort of adult patients treated for aSAH at the Department of Neurosurgery, Skåne University Hospital, Lund, Sweden. This hospital is the sole provider of neurosurgical care for approximately two million inhabitants in southern Sweden. The patients were admitted between November 2014 and March 2018. The study adhered to the Strengthening the Reporting of Observational studies in Epidemiology guidelines (von Elm et al., 2007).

### 2.1 Data variables

During 2014–2018, variables regarding patient- and disease characteristics, treatment, and outcomes were collected in a prospective database. Information was gathered on age, sex, aSAH risk factors, location of the ruptured aneurysm, mode of aneurysm treatment, and whether an external ventricular drain (EVD) was implanted. Neurological status prior to aneurysm occlusion was graded according to the World Federation of Neurosurgical Societies (WFNS) (van Donkelaar et al., 2017) and dichotomized into good grade patients (WFNS I - III) and poor grade patients (WFNS IV - V). Hemorrhage severity on CT was graded according to the Fisher Scale (Fisher et al., 1980). We added further information to the database by reviewing medical records to collect data on the presence of symptomatic DCI. In line with previous studies we defined DCI as a clinical neurological worsening at least three days after the bleeding, where no other clear reasons were found for the deterioration, and narrowing of cerebral vessels was noted on radiological examination (Al-Tamimi et al., 2010).

Information on angiographic vasospasm, and maximum blood flow velocity in the middle cerebral artery (MCA) measured by transcranial doppler (TCD) were registered. Furthermore, we retrospectively obtained additional data on the timing and duration of nimodipine treatment, mode of administration, dosage, administration of vasoactive rescue therapy, symptoms of DCI, and endovascular angioplasty.

### 2.2 Treatment regime and nimodipine administration

Nimodipine was administered enterally, 60 mg every 4 h. In the case of hypotension, 30 mg of nimodipine was administered every 2 h. If hypotension persisted, enteral nimodipine administration was halted, with the option to administer nimodipine intravenously to more finely adjust the dose to avoid lowering the blood pressure. Nimodipine treatment was discontinued after 14 days unless signs and symptoms suggested manifest or impending DCI, regardless of WFNS or Fisher grades. Prolonged treatment was based on increasing TCD velocities, vasospasm on angiography, or clinical symptoms of DCI such as altered state of consciousness, paresis, and dysphasia.

### 2.3 Outcome measures

Functional outcome was graded according to the Glasgow outcome scale extended (GOSE) (Jennett and Bond, 1975). Patients were assessed using a standardized questionnaire (Wilson et al., 1998) one year after the hemorrhage. The functional outcome was dichotomized according to GOSE scores, with 1–4 classified as unfavorable (deceased or in need of assistance during daily activities), and 5–8 as favorable (independent in daily activities). The presence of new infarction on follow-up CT or MRI, later than 30 days post-ictus, that could not be attributed to any other cause was considered radiographic DCI (Wintermark et al., 2006; van der Kleij et al., 2017).

### 2.4 Statistical analyses

All statistical analyses were conducted using SPSS® (IBM Corp. Version 28.0. Armonk, NY, USA). The patient population was divided into two groups; those receiving nimodipine for 14 days or less, and those treated for 15 days or more. As the focus of the present review was length of nimodipine treatment, patients who were not subjected to nimodipine treatment were not included in comparative analysis. Categorical variables were compared using Pearson's chi-squared test, or Fisher's exact test if there were expected cell counts of less than five, and continuous variables using *t*-test if not skewed. Ordinal variables and skewed continuous variables were compared using the Mann-Whitney *U* test. *P*-values less than 0.05 were considered statistically significant.

### 2.5 Ethical considerations

Ethical approval was obtained from the Swedish ethical review authority (reference number 2017/469). Written informed consent was obtained from all patients or their next of kin prior to inclusion in the prospective study database.

## 3 Results

In total, 190 patients were reviewed. Nimodipine was administered to all but two patients who presented to the hospital several weeks after hemorrhage. Twenty-four patients died before post-ictus day 14 for reasons not related to DCI. These patients were not further analyzed. Comparative analysis was based on the remaining 164 patients. Baseline clinical characteristics are presented in Table 1.

**Table 1:** Baseline clinical characteristics.

		≤ 14 days nimodipine (n = 97)	≥ 15 days nimodipine (n = 67)	*p*-value
Age, mean years (SD)		56 (13 %)	55 (13 %)	0.530^a^
Female sex, n (%)		70 (72 %)	47 (70 %)	0.779^b^
Comorbidities, n (%)	Current smoker	42 (43 %)	32 (48 %)	0.693^b^
	Hypertension	34 (35 %)	21 (31 %)	0.621^b^
	Coronary heart disease	6 (6 %)	10 (15 %)	0.064^b^
WFNS grade pre-treatment, n (%)	Good grade (WFNS I - III)	68 (71 %)^d^	28 (44 %)^e^	0.001^b^
	Poor grade (WFNS IV - V)	28 (29 %)^d^	36 (56 %)^e^	
Fisher grade, (%)	1	3 (3 %)^e^	0 (0 %)^e^	0.042^c^
	2	12 (13 %)^e^	2 (3 %)^e^	
	3	36 (38 %)^e^	25 (39 %)^e^	
	4	43 (38 %)^e^	37 (58 %)^e^	
Aneurysm location, n (%)	Anterior circulation	72 (74 %)	59 (88 %)	0.030^b^
	Posterior circulation	25 (26 %)	8 (12 %)	
Aneurysm treatment modality, n (%)	Endovascular	76 (78 %)	49 (73 %)	0.441^b^
	Microsurgical	21 (22 %)	18 (27 %)	
EVD requirement, n (%)		44 (45 %)	50 (75 %)	<0.001^b^

WFNS, World Federation of Neurosurgical Societies; EVD, External Ventricular Drain.

a Independent samples T-test.

b Pearson's chi-squared test.

c Mann-Whitney *U* test.

d One missing value.

e Three missing values.

### 3.1 Nimodipine and DCI

Ninety-seven patients (59 %) received nimodipine for 14 days or less (median 13, IQR 11–14), 67 patients (41 %) received nimodipine for 15 days or more (median 18, IQR 16–21), where15 patients (9 %) received nimodipine for more than 21 days. A majority, 71 %, of patients with good clinical grade had a shorter treatment with nimodipine, whereas the corresponding proportion was 44 % in patients with poor clinical grade aSAH. Length of nimodipine treatment did not differ based on age, sex, comorbidities, or method of aneurysm treatment. However, patients having nimodipine for 15 days or longer more often had poor clinical grade (WFNS IV - V) at admission, higher Fisher grade, more frequently a ruptured aneurysm in the anterior circulation, and more often required an EVD (Table 1). No patient had DCI after nimodipine treatment was discontinued.

The median time between ictus and the first dose of nimodipine was 40 h (IQR 32–54).

Symptomatic DCI occurred in 28 patients (17 %), the median time from ictus to detection was eight days (IQR 6–9) and the most common symptom of DCI was a reduced level of consciousness (54 %) (Table 2). The occurrence of DCI was similar in patients treated with microsurgical clipping (7/39; 18 %) or endovascular aneurysm occlusion (21/125; 17 %), p = 0.8.

**Table 2:** Nimodipine treatment and outcome.

		≤ 14 days nimodipine (n = 97)	≥ 15 days nimodipine (n = 67)	*p*-value
Mode of nimodipine administration, n (%)	po	91 (94 %)	53 (79 %)	0.005^a^
	po + IV	6 (6 %)	14 (20 %)	
Symptomatic DCI, n (%)		5 (5 %)	23 (34 %)	<0.001^a^
	DCI symptom, n (%)			
	Reduced level of consciousness	1 (1 %)	14 (20 %)	<0.001^c^
	Paresis	4 (4 %)	7 (10 %)	0.125^c^
	Dysphasia	0 (0 %)	2 (3 %)	0.165^c^
Days in the neurosurgical department, median (IQR)		14 (11–17)	21 (16–24)	<0.001^b^
Vasoactive rescue treatment, n (%)		2 (2 %)	11 (16 %)	<0.001^c^
Time from ictus to symptomatic DCI, median days (IQR)		8 (6–8)	9 (8–9)	0.478^b^
TCD velocities >120 cm/s, n (%)		17 (18 %)^d^	43 (67 %)^d^	<0.001^a^
Endovascular vasospasm therapy, n (%)		9 (9 %)	31 (46 %)	<0.001^a^
Infarction on follow-up imaging, n (%)		10 (12 %)^f^	14 (24 %)^e^	0.078^a^
Unfavorable functional outcome (GOSE 1–4), n (%)		23 (53 %)	20 (47 %)	0.447^a^

DCI, Delayed Cerebral Ischemia; IQR, Inter-Quartile Range; TCD, Transcranial Doppler.

a Pearson's chi-squared test.

b Mann-Whitney *U* test.

c Fisher's exact test.

d Three missing values.

e Eight missing values.

f Sixteen missing values.

### 3.2 Functional and radiographic outcome

GOSE-questionnaires were completed for 161 patients (98 %), with missing data for 3 patients. Unfavorable outcome (GOSE 1–4) was reported in 43 patients (27 %). The remaining 118 patients (73 %) had favorable outcome (GOSE 5–8). Brain CT or MR imaging later than 30 days post-ictus, evaluated by a neuroradiologist, was available for 140 out of the 161 patients (85 %). Of these, 24 patients (17 %) had developed cerebral infarction assumed to be related to DCI.

## 4 Discussion

The present study reviews the outcome for patients with aSAH after a shorter nimodipine treatment regime than recommended by international guidelines (Diringer et al., 2011; Vivancos et al., 2014; Treggiari et al., 2023; Protocol for Administration of Nimodipine, 2009) in a prospectively collected cohort. In most patients (59 %), nimodipine treatment was discontinued after 14 days or less. The baseline characteristics were similar to those seen in previously published large aSAH cohort studies (Hoh et al., 2023; Wahood et al., 2022; Rinkel and Algra, 2011; Nieuwkamp et al., 2009). More than 40 % of the patients presented with poor clinical grade which differs from previously published studies with short nimodipine treatment regimes (Cho et al., 2016; Sokolowski et al., 2021). The rate of unfavorable functional outcome (27 %) is lower than seen in previously published data (35 55 %) (Rinkel and Algra, 2011; Nieuwkamp et al., 2009; Taufique et al., 2016; van Donkelaar et al., 2019). In the present study, patients that died prior to day 14 post-ictus were excluded, which might influence the rate of unfavorable functional outcome. Importantly, these patients died of causes not related to DCI. The presented rate of DCI (17 %) is in the lower range of what has been published from centers with similar populations and treatment capabilities (17–51 %) (Schmidt et al., 2022; Macdonald, 2014; Roos et al., 2000), suggesting that the current nimodipine treatment regime does not add to poor outcome.

A small number of studies have previously reported the effects of a shortened treatment with nimodipine after aSAH. Sokolowski et al. (2021) presented that patients who received nimodipine for 14 days or less did not experience worse functional outcome compared to those receiving longer treatment. However, the group qualifying for the shorter treatment duration were patients well enough to be discharged 14 days or less post-ictus. Additionally, during a national drug shortage, Cho et al. (2016) also investigated a 14-day nimodipine treatment and found no significant differences in functional outcome compared to the standard 21-day treatment. However, the percentage of patients receiving 14 days of nimodipine was small (18 %), suggesting stricter patient selection. In contrast, we utilize the shorter, 14-day treatment in a wider selection of patients regardless of clinical and radiological grade, prolonging treatment only if symptoms or radiological signs of DCI occur. At our center, patients with DCI are primarily treated with elevation of the blood pressure (usually by norepinephrine): They also receive intravenous fluids (crystalloids, colloids and/or blood transfusions) to avoid hypovolemia and anemia. We follow signs of ischemia by monitoring clinical status and intracerebral microdialysis and the patients are optimized accordingly, sometimes also with restricted mobilization. Endovascular angioplasty can also be effective in treating DCI (Labeyrie et al., 2021) but is only performed if these less invasive measures fail.

A shortened nimodipine treatment duration may benefit the patient and the health care system in several ways. Guidelines recommend that oral nimodipine is administered once every 4 h. In the case of hypotension, half a dose is considered every 2 h (Dorhout Mees et al., 2007; Allen et al., 1983). Therefore, patients need to be woken up several times during the night to receive nimodipine. Frequent administration of medications has been shown to decrease the amount and quality of sleep for patients in the ICU (Gabor et al., 2003). Patients with aSAH have been shown to suffer from sleep disorders in the acute care setting (Fowler et al., 2021) and sleep deprivation has been linked to neurocognitive dysfunction including delirium (Leung et al., 2015; Lowe et al., 2017), impaired immune function (Irwin, 2015) and hyperalgesia (Larson and Carter, 2016) in humans. In rats, sleep deprivation has recently been demonstrated to aggravate brain damage after aSAH (Xu et al., 2021). Therefore, minimizing sleep interruptions is likely beneficial. In addition, potential adverse effects to nimodipine include hypotension, atrial fibrillation, ventricular tachycardia, asystole, gastrointestinal disturbances, and allergic reactions (Chen et al., 2018; Hund et al., 1990). Also, a reduced treatment period will improve the effective utilization of the limited number of beds in neurointensive care units.

Some study strengths and limitations should be noted. The consecutive prospective inclusion of patients in the present study adds to its strength. All patients treated for aSAH at the Department of Neurosurgery at Skåne University Hospital during the given time were reviewed for inclusion. The hospital is the sole provider of neurosurgical care in southern Sweden, minimizing the risk of selection bias. Only three patients were lost to follow-up, giving a follow-up rate of 98 %. As the decision to prolong nimodipine treatment shaped the subgroups based on treatment length the occurrence of DCI cannot be directly compared. The retrospective, observational nature of the study also limited the possibility to adjust for confounding covariates. As factors such as poor WFNS grade is associated with DCI (and thus longer nimodipine treatment), the groups (reduced and normal length of nimodipine treatment) are not independent but are created according to patient characteristics and clinical course. Therefore, the results have to be interpreted with the risk of influence by confounding by indication.

Although the occurrence of poor functional outcome did not differ based on treatment length, we cannot preclude that a longer treatment regime would even further improve the results of patients having received a shorter treatment.

A large randomized study is required to answer the question whether a shorter treatment with nimodipine is enough to give full benefit of the medication in patients without signs and symptoms of DCI. An ad hoc analysis on health economics could also clarify the benefit of this treatment regimen.

## 5 Conclusion

A shorter nimodipine treatment in patients not exhibiting signs and symptoms of DCI after aSAH corresponds with functional outcome rates similar to previously published studies on the full-length treatment regimen. No readmissions or signs of DCI were seen after discontinuation of nimodipine.

## Declaration of competing interest

The authors of this manuscript, *Nimodipine after aneurysmal subarachnoid hemorrhage: shortened treatment in an unselected patient cohort*, have no competing interest.
